# Evidence for Progressive Cognitive Deficits in Patients With Major Depressive Disorder

**DOI:** 10.3389/fpsyt.2021.627695

**Published:** 2021-02-16

**Authors:** Jin Liu, Bangshan Liu, Mi Wang, Yumeng Ju, Qiangli Dong, Xiaowen Lu, Jinrong Sun, Liang Zhang, Hua Guo, Futao Zhao, Weihui Li, Li Zhang, Zexuan Li, Yan Zhang, Mei Liao, Lingjiang Li

**Affiliations:** ^1^Department of Psychiatry, The Second Xiangya Hospital, Central South University, Changsha, China; ^2^Hunan Key Laboratory of Psychiatry and Mental Health, China National Clinical Research Center on Mental Disorders (Xiangya), China National Technology Institute on Mental Disorders, Hunan Technology Institute of Psychiatry, Mental Health Institute of Central South University, Changsha, China; ^3^Zhumadian Psychiatric Hospital, Zhumadian, China

**Keywords:** major depressive disorder, cognitive deficit, progression, recurrent depression, the number of episodes, executive function, shifting

## Abstract

**Background:** Cognitive deficits have shown progressive feature in major depressive disorder (MDD). However, it remains unknown which component of cognitive function is progressively impaired across episodes of MDD. Here we aim to identify the progressively impaired cognitive components in patients with MDD.

**Methods:** A comprehensive neurocognitive test battery was used to assess the cognitive components (executive function, attention, processing speed, memory, working memory, inhibition, shifting, and verbal fluency) in 35 patients with first-episode MDD (FED), 60 patients with recurrent MDD (RD) and 111 matched healthy controls (HCs). After 6 months of treatment with antidepressant, 20 FED and 36 RD patients achieved clinical remission and completed their second-time neurocognitive tests. Statistical analyses were conducted to identify the impaired cognitive components in the FED and RD groups before and after treatment, and to assess the relationship between the cognitive components and the number of episodes and total illness duration in the MDD patient group.

**Results:** At baseline, both the FED and RD groups showed impairments in all of the cognitive components; the FED and RD groups showed no significant difference in all of the components except for shifting. After remission, only shifting in the RD group showed no significant improvement and remained in an impaired status. Furthermore, shifting was the only component negatively correlated with the number of episodes as well as the total illness duration.

**Conclusions:** Shifting may serve as the progressive cognitive deficit across episodes of MDD.

**Clinical Trials Registration:** Registry name: HPA function and MRI study of trauma-related depression; Registration number: ChiCTR1800014591; URL: http://www.chictr.org.cn/edit.aspx?pid=24669&htm=4.

## Introduction

Major depressive disorder (MDD) is a debilitating mental disorder characterized by life-long recurring episodes. Among patients who achieved full remission after a major depressive episode, 16% will develop at least one new episode in the next 6 months, 26% will develop an episode in 1 year (1), and 85% will have that experience again in 15 years (2). According to some studies, the increasing number of episodes is positively correlated with increasing severity of symptoms, longer duration of illness, increasing vulnerability of developing new episodes, and increasing risk of relapse (3–5), suggesting a progressive nature of depression pathology. The progressive nature of MDD may be a major cause of its poor prognosis and chronicity, which have been reported to be associated with impairment in social function and high disease burden (6–9).

It is well-established that cognitive deficits, mainly involving executive function, attention, processing speed, and memory (10, 11), are currently acknowledged as a most pronounced clinical feature of MDD (12–14). Progressive cognitive deficits are the deficits deteriorating with increasing number of episodes and cumulative length of illness duration. Given the enduring and accumulating characteristics of progressive deficits of MDD, it is noteworthy that the progressive cognitive deficits tend to develop into residual cognitive symptoms even when patients have achieved clinical remission, which has been repeatedly reported to be associated with poor prognosis and a higher relapse rate (5, 15–17). Therefore, the identification of the progressive cognitive deficits will not only provide new insight into the chronicity of MDD, but will also help in developing therapeutic strategy to treat residual cognitive symptoms in MDD.

Although many studies have investigated various cognitive features in MDD, only a small number of studies addressed the progressive deteriorating characteristic of cognitive deficits related to this condition. Some previous cross-sectional studies revealed deficits in different cognitive domains in remitted patients, which were associated with prior episodes (18–21). Talarowska et al. (22) reported that patients with recurrent depression (RD) performed worse in tasks on executive function, processing speed, visual-spatial and auditory-verbal memory than patients with first-episode depression (FED), while Roca et al. (23) found no difference in overall cognitive tests between FED and RD patients, even when they were experiencing an acute episode. An earlier longitudinal study by Maeshima et al. (24) found that memory deficits in remitted FED patients disappeared after 3 years of remission, while still persisted in remitted RD patients. Although there is emerging evidence indicating that there might be potential progressive cognitive deficits in MDD, most of those studies had small sample size, were cross-sectional designed, or only assessed one or a small number of cognitive domains, resulting in controversies and insufficient information to identify the cognitive deficits with clinical progressive characteristics. Specifically, it is still unclear which cognitive deficits will deteriorate with increasing episodes and whether these cognitive deficits are the result of enduring and accumulating effects of previous depressive episodes.

Using a comprehensive battery of neurocognitive tests in 35 FED, 60 RD patients and 111 matched healthy controls (HC), the present study aims to investigate the progressive cognitive deficit in MDD. Dual analytic strategies were adopted: firstly, we analyzed the differences in cognitive performance between the FED and RD patients under their episode and remission phases, respectively; and the changes in the patients' cognitive performance across 6 months of treatment were also observed simultaneously. Secondly, we analyzed the correlation between the cognitive performance and the number of episodes as well as the total illness duration. We hypothesized that the cognitive deficit with progressive characteristics might result from the aberrance of the cognitive domain which was associated with poorer performance in RD patients than FED patients both under the acute episode and the remission phase. Moreover, the improvement of progressive cognitive component would fall behind other depression-related symptoms in time and degree and might deteriorate with increasing episodes.

## Methods and Materials

### Participants

One hundred two medication-free MDD patients and 111 HCs were recruited from the Zhumadian Psychiatric Hospital (Henan, China) and its nearby communities from 2013 to 2018. All the MDD patients met the following inclusion criteria: (1) 18–60 years old, with an education of ≥6 years, and right-handed; (2) meet the criteria for MDD in the Structured Clinical Interview for DSM-IV (SCID-IV); (3) with a current episode of depression for at least 2 weeks, indicated by a 24-item Hamilton Depression Rating Scale (HAM-D_24_) score ≥20; (4) with no psychotropic drug use for over 2 weeks (6 weeks for fluoxetine) before recruitment. Detailed inclusion criteria and exclusion criteria for the MDD and HC groups were reported in our previous studies (25, 26).

This study was approved by the medical ethics committees of the Second Xiangya Hospital of Central South University and the Zhumadian Psychiatric Hospital. Written informed consent form was obtained from all the participants.

### Treatment and Efficacy Assessment

All the subjects received the clinical assessment and neurocognitive tests at baseline, and MDD patients underwent all the neurocognitive tests again after 6 months of treatment with paroxetine. For all the MDD patients, clinical symptoms were assessed using the HAM-D_24_ at the end of the 0.5, 1st, 2nd, 3rd, 4th, 5th and 6th months. Clinical remission was considered when a patient achieved a HAM-D_24_ score of ≤ 7, which lasted for at least 2 months. During the treatment period, each patient received 10 mg of paroxetine in the first week and 20 mg or higher in the following weeks, depending on their treatment response and side effects. The maximum dose was 60 mg. Of the 102 patients initially enrolled, 7 had manic onset during the treatment, and thus were excluded. Therefore, 95 patients (35 with FED and 60 with RD) were included for the baseline analysis. Additionally, 7 patients received electroconvulsive therapy or other antidepressant drugs, and 25 patients withdrew from the study. Therefore, 63 patients completed the 6 months of treatment; among them, 56 patients (20 with FED and 36 with RD) who achieved clinical remission were finally included for post-treatment analyses, while 7 unremitted patients were not included because of the unproportionally small sample size.

### Neurocognitive Assessment

All the participants underwent a comprehensive battery of neurocognitive tests involving executive function, attention, processing speed, and memory (25, 26). The standardized Z-scores of different tests were summed up to form cognitive domains. The internal consistency of different tests in each cognitive domain was assessed using Cronbach's alpha.

#### Executive Function

The digit span backward test in the Wechsler Adult Intelligence Scale-Revised (WAIS-R), the color-word interference condition of the Stroop test, the Trail-Making Test part B (TMT-B), and the semantic Verbal Fluency (animals) test were used to assess the four subcomponents of executive function, i.e., working memory, inhibition, shifting, and verbal fluency, respectively (27). It was notable that the patients who performed significantly poorly in TMT-B always needed more time to complete the Trail Making Task part A (TMT-A). The final performance of TMT-B was affected by the performance of TMT-A because the performance of TMT-A reflects the initial processing speed. The shifting components were obtained by subtracting the TMT-A result from the TMT-B result (28) (Cronbach's alpha 0.616).

#### Attention

The Stroop Word Test and the digit span forward test were used to assess the subjects' sustained attention (27) (Cronbach's alpha 0.576).

#### Processing Speed

TMT-A and the Stroop Color Test were used to assess the subjects' processing speed (29) (Cronbach's alpha 0.597).

#### Memory

Three tests from the Wechsler memory scale, i.e., the Visual Memory Test, the Intelligent Memory Test and the Associative Memory Test, were used to assess the subjects' visual memory, intelligent memory, and associative memory, respectively (30) (Cronbach's alpha 0.718).

### Statistical Analysis

In this study, the average z-score per subtest for each domain was calculated to level out the difference in the number of subtests across domains (25). Z-scores were calculated using the equation X_individual_-$\bar{\text{X}}$_controls_/σ_controls_. X_individual_ represents the raw scores of each individual, X_controls_ represents the mean value of the controls, and σ_controls_ refers to the standard deviation of the controls. Higher Z-scores indicated better performance for all variables, and this value should be reversed when lower values indicated better performance (in TMT-A and TMT-B). The normality of the variables was tested using Kolmogorov–Smirnov test. Log or square root transformations were used for skewed variables to achieve normality.

One-way analyses of variance (ANOVA) was performed to make sure that the age and education were comparable between the FED, RD, and HC groups, and between the remitted FED (rFED), remitted RD (rRD) and HC groups. Two-sample *t*-test was used to compare the HAM-D_24_ score, total illness duration and the number of episodes between the FED and RD groups, and the rFED and rRD groups. Chi-square test was used to ensure that the gender ratio of the groups was matched. Multivariate analysis of covariance (MANCOVA) was conducted to compare the overall cognitive domain between the FED, RD and HC groups, and between the rFED, rRD, and HC groups, with age, gender and education as covariates. Paired *t*-test was used to compare the test results between pre-treatment and remission phases for both FED and RD patients. The significance level (two-tailed) was set at *p* < 0.05. All statistical analyses were conducted using SPSS 24.0.

## Results

### Demographic and Clinical Characteristics

Table 1 showed the demographic and clinical characteristics of the MDD (FED and RD), rMDD (rFED and rRD), and HC groups. There were no inter-group differences between the FED, RD and HC groups, and between the rFED, rRD, and HC groups regarding age, gender, and education. And there were also no significant differences in HAM-D_24_ total score between the FED and RD groups, and between the rFED and rRD groups.

**Table 1:** The demographic and clinical characteristics of the MDD, rMDD, and HC groups.

	**MDD** ** (m** **±** **s.d.)**		**rMDD** ** (m** **±** **s.d.)**		**HC** ** (m ± s.d.)**	***p_**1**_***	***p_**2**_***
	**FED**	**RD**	**rFED**	**rRD**			
Age (years)	32.11 ± 8.42	34.82 ± 8.84	32.85 ± 9.33	36.44 ± 9.20	34.85 ± 8.90	0.25^a^	0.35^a^
Gender (F/M)	17/18	38/22	9/11	23/13	59/52	0.30^b^	0.35^b^
Education (years)	11.23 ± 3.47	10.25 ± 3.34	11.45 ± 3.59	10.44 ± 3.42	10.78 ± 3.24	0.34^a^	0.56^a^
HAM-D_24_	34.89 ± 7.47	32.40 ± 6.80	2.71 ± 2.02	3 ± 2.20	–	0.11^c^	0.64^c^
Total illness duration (months)	3.63 ± 2.22	66.13 ± 53.75	6.73 ± 2.13	70.94 ± 59.44	–	** <0.001**^c^	** <0.001**^c^
Number of episodes	1 ± 0	2.83 ± 1.52	1 ± 0	2.77 ± 1.68	–	** <0.001**^c^	** <0.001**^c^

*MDD, major depressive disorder (current episode); FED, first-episode depression; RD, recurrent depression; HC, healthy control; rMDD, remitted depression*.

a *ANOVA*,

b *Chi-square test*,

c *Two-sample t-tests*.

*p_1_ values denote the statistical result between the FED, RD, and HC groups*.

*p_2_ values denote the statistical result between the rFED, rRD, and HC groups*.

*Bold values indicate statistical significance*.

### Cognitive Performance Between FED, RD, and HC Group

MANCOVA analyses revealed significant overall differences between the FED, RD, and HC groups in all the four cognitive domains (*F* = 10.60, *p* < 0.001; see Table 2), with age, gender and education as covariates. *Post-hoc* comparisons confirmed that both the FED and RD groups performed significantly more poorly in all the four cognitive domains, compared with the HC group (all *p* < 0.01). However, no significant differences were found between the FED and RD groups in all the four cognitive domains (all *p* > 0.05).

**Table 2:** Cognitive Performance (Z-score) of the FED and RD patients in episode and remission phases (MANCOVA test).

	**MDD**		**rMDD**		**HC**	***p_**1**_***	***p_**2**_***
	**FED**	**RD**	**rFED**	**rRD**			
Executive function	−0.31 ± 0.69^***^	−0.71 ± 0.79^***^	−0.10 ± 0.63	−0.35 ± 0.86	0.00 ± 0.71	** <0.001**	**0.025**
Working memory	−0.31 ± 0.95^**^	−0.46 ± 0.73^**^	0.00 ± 0.66	−0.10 ± 0.80	0.00 ± 1.00	**0.001**	0.596
Inhibition	−0.48 ± 0.92^**^	−0.49 ± 1.09^**^	0.18 ± 0.97	−0.33 ± 0.97	0.00 ± 1.00	**0.001**	0.345
Shifting	0.10 ± 1.64	−1.12 ± 2.08^***^^ΔΔ^	−0.22 ± 1.45	−0.68 ± 1.69^**^	0.00 ± 1.00	** <0.001**	**0.020**
Verbal fluency	−0.55 ± 0.73^***^	−0.75 ± 0.73^***^	−0.37 ± 0.69^*^	−0.28 ± 0.77	0.00 ± 1.00	** <0.001**	**0.043**
Attention	−0.40 ± 0.95^***^	−0.56 ± 0.84^***^	0.08 ± 0.83	−0.31 ± 0.93	0.00 ± 0.83	** <0.001**	0.313
Processing speed	−0.99 ± 1.20^***^	−0.81 ± 0.85^***^	0.09 ± 0.57	−0.27 ± 0.95	0.00 ± 0.84	** <0.001**	0.401
Memory	−0.65 ± 0.97^***^	−0.69 ± 1.07^***^	0.33 ± 0.65	0.14 ± 0.72	0.00 ± 0.78	** <0.001**	0.056

*MDD, major depressive disorder (current episode); FED, first-episode depression; RD, recurrent depression; HC, healthy control; rMDD, remitted depression*.

*p_1_-values denote the statistical result between the FED, RD, and HC groups*.

*p_2_-values denote the statistical result between the rFED, rRD, and HC groups*.

* *p < 0.05*,

** *p < 0.01*,

*** *p < 0.001 vs. HC*;

ΔΔ *p < 0.01 vs. FED*.

*Bold values indicate statistical significance*.

Although there was no significant statistical difference between the FED and RD groups in term of executive function, the gap in the mean value between these two groups was relatively large (−0.31 ± 0.69 vs. −0.71 ± 0.79, *p* = 0.088). Therefore, we further investigated four core sub-components of executive function. The MANCOVA analyses revealed significant overall differences between the FED, RD, and HC groups in all the four sub-components of executive function (*F* = 7.82, *p* < 0.001; see Table 2), with age, gender and education as covariates. *Post-hoc* comparisons confirmed that both the FED and RD groups performed significantly more poorly in all the four sub-components of executive function, compared with the HC group (all *p* < 0.01). However, a significant difference was only found between the FED and RD groups regarding the shifting component (*p* < 0.01), while no significant differences were found regarding working memory, inhibition and verbal fluency (all *p* > 0.05).

### Cognitive Performance Across Episodes and Remission States

Fifty six patients achieved clinical remission at the end of the 6th month. Paired *t*-test was used to compare the test results between the pre-treatment and remission phases for both the FED and RD groups, respectively (Table 3). For the FED group, significant improvements were found in attention, processing speed and memory (*p* < 0.05), while no significant improvement was noticed in executive function (*p* = 0.055) after the 6-month treatment period. Among the four sub-components of executive function, significant improvements were found in working memory (*p* = 0.048) and inhibition (*p* < 0.001), while no significant improvement was found in the shifting component (*p* = 0.886) and verbal fluency (*p* = 0.404).

**Table 3:** Cognitive Performance (Z-score) of the FED and RD patients in episode and remission phases.

	**rFED-BS**	**rFED**	***t***	***p_**1**_***	**rRD-BS**	**rRD**	***t***	***p_**2**_***
Executive function	−0.38 ± 0.67	−0.10 ± 0.63	−2.05	0.055	−0.71 ± 0.86	−0.35 ± 0.86	−3.81	**0.001**
Working memory	−0.39 ± 0.93	0.00 ± 0.66	−2.12	**0.048**	−0.46 ± 0.66	−0.10 ± 0.80	−3.17	**0.003**
Inhibition	−0.46 ± 1.03	−0.17 ± 0.97	−5.70	** <0.000**	−0.47 ± 1.09	−0.33 ± 0.97	−0.95	0.349
Shifting	−0.15 ± 1.29	−0.22 ± 1.45	0.15	0.886	−1.26 ± 2.42	−0.68 ± 1.69	−1.74	0.092
Verbal fluency	−0.53 ± 0.73	−0.37 ± 0.69	−0.85	0.404	−0.63 ± 0.74	−0.28 ± 0.77	−3.21	**0.003**
Attention	−0.36 ± 1.09	0.08 ± 0.83	−2.36	**0.030**	−0.52 ± 0.86	−0.31 ± 0.93	−2.29	**0.028**
Processing speed	−1.00 ± 1.23	0.09 ± 0.57	−5.12	** <0.001**	−0.97 ± 0.89	−0.27 ± 0.95	−8.54	** <0.001**
Memory	−0.65 ± 0.92	0.33 ± 0.65	−4.52	** <0.001**	−0.68 ± 1.10	0.14 ± 0.72	−6.33	** <0.001**

*rFED, remitted first-episode depression; rFED-BS, remitted first-episode depression (at baseline); rRD, remitted recurrent depression; rRD-BS, remitted recurrent depression (at baseline)*.

*p_1_-values denote the paired t-test result between the rFED-BS and rFED groups*.

*p_2_-values denote the paired t-test result between the rRD-BS and rRD groups*.

*Bold values indicate statistical significance*.

In the RD group, significant improvements were found in all the four cognitive domains after 6 months of treatment. Among the four sub-components of executive function, significant improvements were found in working memory (*p* = 0.003) and verbal fluency (*p* = 0.003), while no significant improvement was found in the shifting component (*p* = 0.092) and inhibition (*p* = 0.349).

### Cognitive Performance of the rFED, rRD, and HC Groups

MANCOVA analyses revealed significant overall differences between the three groups in all the four cognitive domains (*F* = 2.88, *p* = 0.04, see Table 2) and all the four sub-components of executive function (*F* = 1.99, *p* = 0.047, see Table 2), with age, gender and education as covariates. *Post-hoc* comparisons showed no significant difference in the performance regarding all the four cognitive domains between the rFED, rRD, and HC groups (all *p* > 0.05). Among the four sub-components of executive function, only the performance of verbal fluency in the rFED group (*p* = 0.043) and the shifting component in the rRD group (*p* = 0.02) were significantly worse than the HCs. No significant difference was found between the rFED and rRD groups in all the four cognitive domains and four sub-components of executive function (all *p* > 0.05).

### Correlation Between Cognitive Performance and the Number of Episodes as Well as Total Illness Duration in MDD Patients

**Figure 1:**
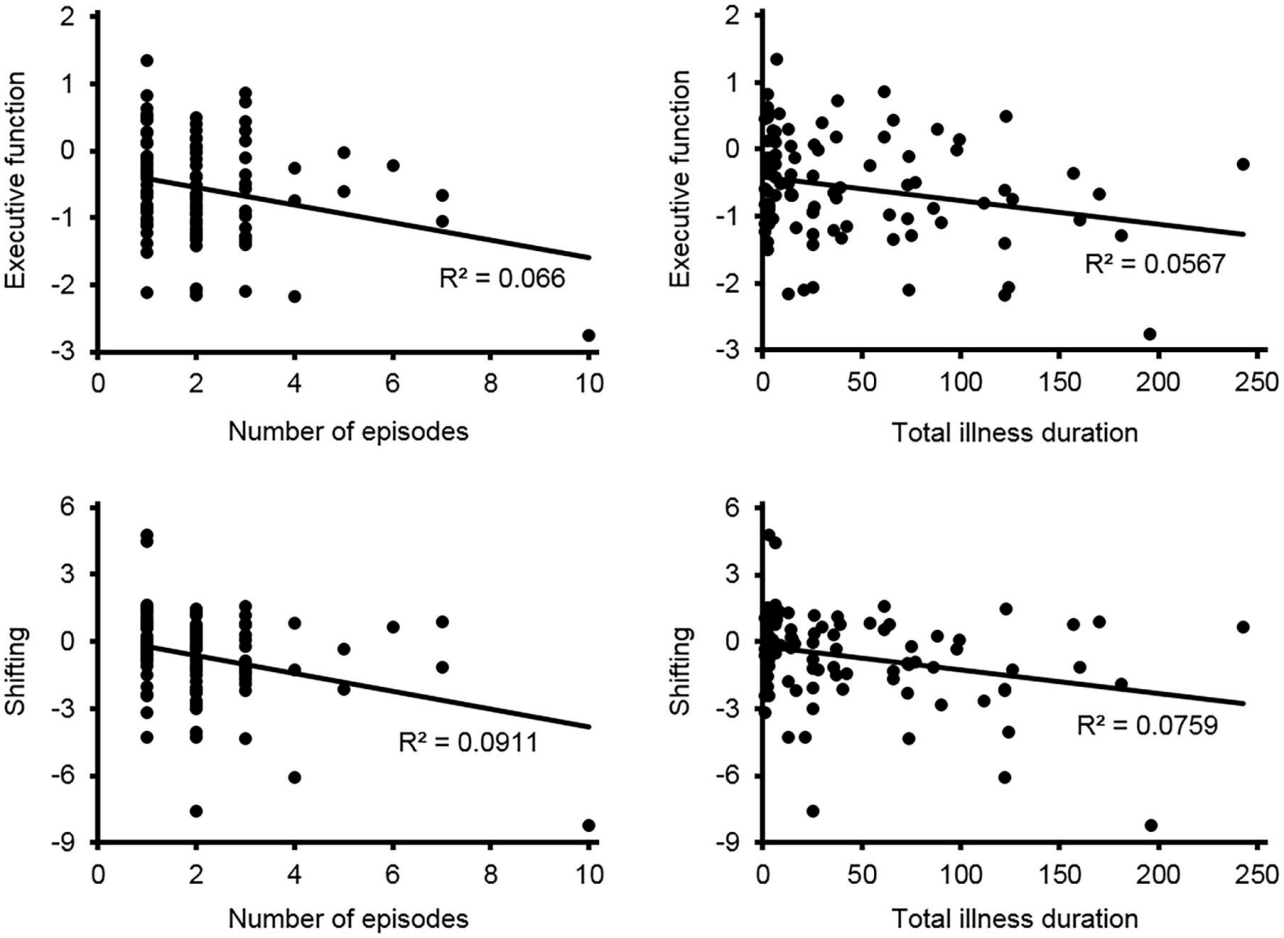
The correlation between cognitive performance and the number of episodes as well as total illness duration in patients with MDD.

Pearson correlation analyses were conducted to reveal the relationships between the cognitive performance and the number of episodes as well as total illness duration in MDD patients. Among all the cognitive domains, only a negative correlation between executive function and the number of episodes (*r* = −0.257, *p* = 0.012) and total illness duration (*r* = −0.238, *p* = 0.020), and a negative correlation between the shifting component and the number of episodes (*r* = −0.302, *p* = 0.003) and total illness duration (*r* = −0.276, *p* = 0.007) were found (see Figure 1).

## Discussion

Using a comprehensive cognitive assessment battery, we have investigated the cognitive deficit with progressive characteristics in MDD in the present study. To our knowledge, this is the first study providing two critical pieces of evidence to identify the progressive cognitive deficit across the course of depression. The main outcome was that both the FED and RD groups showed impairments in all of the cognitive components, compared with the HC group, but there was no significant difference in all of the components except for one sub-component of executive function, shifting, between the two patient groups. After remission, only the shifting component in the RD group showed no significant improvement and remained in an impaired status. Notably, the shifting component was the only component negatively correlated with increasing episodes and cumulated illness duration. These results are consistent and revealed that the chronicity of depression exerts an extended and cumulative effect on shifting.

Executive function is a significant component found through the analyses. Although no significant difference was found between the FED and RD groups in all the four cognitive domains, the domain of executive function showed a notable gap in the mean value between these two groups. In addition, correlation analyses showed that executive function was negatively correlated with the number of episodes as well as total illness duration. These findings indicated that among these four cognitive domains, executive dysfunction might be the impairment most likely to be progressive in MDD. Being the most common and prominent cognitive deficit in MDD, executive dysfunction mainly comprises of four sub-components: working memory, inhibition, shifting and verbal fluency (27). Previous studies have proposed that these four sub-components were well-separated and the impairment of each of them had unique patterns (14, 27). Therefore, we further investigated these four sub-components of executive function and found that the impairment of shifting was most significant in the aspect of progressive feature.

Shifting is defined as the ability to respond to the switching between different tasks or situations and to adapt to changing demands (31). Involved in a variety of cognitive processes, such as working memory, inhibition, and attention, shifting is considered a superior and more comprehensive subcomponent of executive function (32). The wide involvement of shifting might be one of the reasons why the improvement of shifting impairment falls behind other depression-related symptoms, even when the MDD patient has achieved clinical remission. Our findings are in line with previous studies, which reported the impairment in shifting both in acute and in remitted MDD patients (14, 27). As an extension of previous findings, our results also suggest that the impairment in shifting might deteriorate with increasing number of episodes; presumably, the impairment in shifting might not be a state-like deficit, and thus might improve slower and to a lesser degree, compared with those state-related symptoms. Taken together, the evidence provided further insight into the dynamic shifting impairment process following a major depressive episode. Accumulating with future depressive episodes, such shifting impairment might become persistent and progressive.

The neurobiological basis of MDD is essential for us to understand the progressive nature of cognitive deficits. Previous neuroimaging findings reported that widespread structural and functional alterations were associated with the number of episodes and influenced by cumulated illness durations (33–35). Besides, Yan et al. found that patients with RD showed more severe functional connectivity disruption and more extensive functional connectivity network abnormality than those with FED (36). These findings provided evidence for the progressive feature of depression across the course of the illness. In addition, structural and functional alterations in the thalamus, anterior cingulate cortex, putamen, and prefrontal cortex were associated with shifting (37, 38), and the alterations in these brain regions have been reported to be associated with the number of episodes and the cumulated illness durations (33, 39–42). Therefore, it is conceivable that shifting is progressively impaired along with the disruption of the related brain regions.

Widespread cognitive deficits were detected in all the four cognitive domains and all the four subcomponents of executive function at the acute episode phase. After 6 months of treatment, significant improvement of attention, speed of processing, memory, and working memory was found in the rFED and rRD patients, while no significant difference was found between the remitted MDD patients and HCs in all the cognitive domains except for shifting in the rRD patients and except for verbal fluency in the rFED patients. There is a number of previous studies indicating that the improvement of these cognitive performances is state-related and the relation between clinical remission and these cognitive domains is remarkable (23, 43). Additionally, no statistical difference was found in all cognitive domains except for shifting between the FED and RD patients both in the acute and remitted phases. These results were in line with the findings of Roca et al. (23), but inconsistent with the study by Talarowska et al. (22), which reported that patients with RD performed more poorly than those with FED in various cognitive domains during depression episode. We presume that the reason for the inconsistency mentioned above might be the significantly older age of the RD patients than the FED patients in that study, and age is generally considered as a crucial risk factor for cognitive performance (29, 44–47). We have also found that the performance related to these cognitive domains (except for executive function and shifting) was not associated with the number of episodes and total illness duration, which provided another convincing piece of evidence that the impairment in these cognitive domains might be state-related.

It is notable that verbal fluency in patients with rFED was worse than HCs, while no statistical difference was found between the rRD patients and HCs. The association between clinical remission and verbal fluency was obvious in patients with RD while unapparent in those with FED. Similar results were also found by Roca et al. (23). They speculated that patients with rFED performed worse in shifting than those with rRD may be due to the greater relevance of cognitive bias in onset of FED patients rather than RD patients. Besides, it is possible that patients with RD have developed more effective compensatory mechanisms for major depressive episode. Previous studies suggested that clinical remission was associated with the compensation (48). The compensatory mechanisms might be more thorough as the illness progress. Nonetheless, we could not rule out the possibility that the small sample size of the rFED group affected the power of statistical analyses. To further clarify the association between clinical remission and verbal fluency in FED and RD patients, prospective design is essential in future works.

Strengthened by the longitudinal design in a cohort of medication-free MDD patients, the present study still has several limitations. Firstly, a relevant number of patients dropped out during the 6-month treatment course due to unavoidable reasons (such as poor response to paroxetine, severe gastrointestinal reactions, inconvenience of visiting the site, etc.). However, there are no statistically significant differences in demographic and clinical features in baseline between remitted patients and other participants (non-remitted patients and dropouts). The relatively high dropout rate resulted in a quite small number of non-remitted patients included in this study, which limited the exploration of the change trajectory of cognitive performance under the episode, non-remission, and remission states. Secondly, the neurocognitive performance of the HCs was assessed only once at baseline, which limited the time × group statistical comparison. Therefore, we could not rule out the possible effects of time and test-retest practice in this study. Thirdly, the small sample size, especially for the rFED group, might have affected the statistical power and increased the chance of type II errors. Fourthly, the correlation analyses of the association between cognitive performance and the number of episodes as well as total illness duration have made it difficult to make causal inferences. According to our inference, the possibility that poor shifting ability increases relapse rate cannot be ruled out, which is also supported by the evidence for progressive feature of the impairment in cognitive performance. Finally, recurrent patients had used different antidepressants before entering this study. To eliminate the influence of previous antidepressant use, we only enrolled patients who were not taking psychotropic drugs for at least 2 weeks (6 weeks for fluoxetine) before inclusion.

In conclusion, to our knowledge, the present study has, for the first time, investigated the progressive cognitive deficit of MDD by combining the evidence of cognitive performance in different phases of the disease and the association with the number of episodes in a relatively large cohort. The results provided new evidence supporting the progressive characteristics of shifting impairment across the course of depression. Besides, the results also provided moderate evidence for the state-related feature of attention, processing speed, memory, and working memory impairments in MDD. These findings not only provide evidence to understand the progressive feature of cognitive impairments, but also help in the development of therapeutic strategy for residual cognitive symptoms in MDD. Future studies with a longer period, especially longitudinal studies including different phases of the disease are needed to further identify the state-related, trait-related and progressive features of cognitive impairments.

## Data Availability Statement

The original contributions presented in the study are included in the article/supplementary material, further inquiries can be directed to the corresponding author/s.

## Ethics Statement

The studies involving human participants were reviewed and approved by the medical ethics committees of the Second Xiangya Hospital of Central South University and the Zhumadian Psychiatric Hospital. The patients/participants provided their written informed consent to participate in this study.

## Author Contributions

JL: conceptualization, data curation, formal analysis, investigation, writing–original draft, and writing–review & editing. BL: data curation, investigation, and writing–review & editing. MW, YJ, QD, XL, JS, LiaZ, HG, and FZ: data curation and investigation. WL, LiZ, and ZL: investigation. YZ: investigation and writing–review & editing. ML: conceptualization, funding acquisition, methodology, project administration, supervision, and writing–review & editing. LL: conceptualization, funding acquisition, investigation, methodology, project administration, supervision, and writing–review & editing. All authors contributed to the article and approved the submitted version.

## Conflict of Interest

The authors declare that the research was conducted in the absence of any commercial or financial relationships that could be construed as a potential conflict of interest.
